# Post-traumatic stress disorder affects fucose-α(1–2)-glycans in the human brain: preliminary findings of neuro deregulation using in vivo two-dimensional neuro MR spectroscopy

**DOI:** 10.1038/s41398-018-0365-6

**Published:** 2019-01-18

**Authors:** Scott Quadrelli, Nathan Tosh, Aaron Urquhart, Katie Trickey, Rosanna Tremewan, Graham Galloway, Lisa Rich, Rodney Lea, Peter Malycha, Carolyn Mountford

**Affiliations:** 1 Translational Research Institute, Woolloongabba, QLD 4024 Australia; 2 Center for MR in Health, University of Newcastle, Newcastle, NSW 2308 Australia; 3 Institute of Health and Biomedical Innovation, Queensland University of Technology, Brisbane, QLD 4000 Australia; 4 Radiology Department, Princess Alexandra Hospital, Woolloongabba, QLD 4024 Australia

## Abstract

Post-traumatic stress disorder (PTSD) is triggered by experiencing terrifying event(s) for which there is currently no objective test for a definitive diagnosis. We report a pilot study where two-dimensional (2D) neuro magnetic resonance spectroscopy (MRS), collected at 3 T in a clinical scanner with a 64-channel head coil, identifies neuro deregulation in the PTSD cohort. The control subjects (*n* = 10) were compared with PTSD participants with minimal co-morbidities (*n* = 10). The 2D MRS identified statistically significant increases in the total spectral region containing both free substrate fucose and fucosylated glycans of 31% (*P* = 0.0013), two of multiple fucosylated glycans (Fuc IV and VI) were elevated by 48% (*P* = 0.002), and 41% (*P* = 0.02), respectively, imidazole was increased by 12% (*P* = 0.002), and lipid saturation was increased by 12.5% (*P* = 0.009). This is the first evidence of fucosylated glycans, reported in animals to be involved in learning and memory, to be affected in humans with PTSD.

## Introduction

Post-traumatic stress disorder (PTSD) is a mental health condition precipitated by experiencing or witnessing a terrifying event(s). Symptoms of PTSD generally include re-experiencing of the trauma event (flashbacks, intrusive memories), significant avoidance of triggers, marked alterations in mood/beliefs, and hyperarousal (including panic/hypervigilance)^1^. There is an alarmingly high incidence of PTSD in the defense forces and front-line defenders. PTSD is a common mental health condition, with an estimated 12 month prevalence of 5.2% vs. 8.3% in the general and military populations, respectively^2^. A survey of more than 4000 first responders found that 6.6% had attempted suicide, which is more than 10 times the rate in the general population^3^. As yet there is no objective test for this condition and diagnosis relies on a series of psychological evaluations as described below in methods.

Evaluation of human neurochemistry can be achieved by undertaking in vivo one-dimensional (1D) and more recently two-dimensional (2D) MR spectroscopy^4,5^. This technology has been used to provide the first objective evaluation of chronic pain^6^ and repetitive head injury^7,8^.

Others have used 1D MRS to evaluate the PTSD. Karl and Werner^9^ reported, using a meta-analysis, reduced NAA/Cr ratio in the anterior cingulate cortex (ACC) and hippocampi. There is a report of glutamatergic dysfunction and deregulation of GABA in some brain regions associated with expected hypo-activation in PTSD. In this latter case, the choice of voxel location was determined by fMRI^10–12^.

One dimensional (1D) MR spectroscopy, evaluated by conventional means of resonance frequency and intensity, has considerable limitations due to the overlapping of chemical species in common spectral regions. A way to overcome this limitation is the use of 2D in vivo neuro spectroscopy, specifically 2D COrrelated SpectroscopY (COSY)^4,13–15^ which allows unambiguous assignment of metabolites, lipids and glycans as well as offering the opportunity to more accurately measure the size of the difference when compared with a healthy cohort.

The in vivo 2D L-COSY technology was historically developed by studying the posterior cingulate gyrus (PCG); as at that time in the development of the technology, it generated the strongest signal due to proximity to the coil. This is no longer the case, with the increase from 8- to 64-channel head coils, more stable magnets and better water suppression now in use in clinical scanner. Nevertheless, it remains the region of the brain where most 2D L-COSY studies, from a range of conditions including chronic pain, multiple sclerosis, and blast exposure, are available for comparison.

The PCG region is part of the posteromedial cortex and is a highly connected and metabolically active region and has been invoked in Alzheimer’s and traumatic brain injury^7,16,17^. The PCG is thought to link the hippocampal formation and the higher-level cortices, and may have a role in the balance between internal and external thought^18^, this region is also a key component of the default mode network, which demonstrates reduced connectivity in PTSD^19^. Also, the PCG is reported to be responsive to emotional stimuli and hypothesized to integrate stimuli with higher order processing^20,21^.

New neurochemical assignments have been made possible using the 2D-COSY protocol with the latest hardware capabilities. These include multiple fucose-α(1–2)-glycans and the substrate α fucose in the spectral region F2, 3.95–4.50 ppm; F1, 0.90–1.70 ppm^15^. From animal studies, a growing body of literature implicates, fucose-α(1–2)-glycans, the molecular mechanisms that underlie neuronal development, learning, and memory in the brain^22–26^. The fucose-α(1–3)-glycans reside under the water signal and are not yet available for inspection.

It was our hypotheses that the in vivo neuro 2D L-COSY protocol would identify the molecular species that are deregulated in the human brain in those with PTSD, as well as providing an insight into whether the fucose-α(1–2)-glycans are affected by this condition. The goal was to generate an objective test for PTSD and evidence of deregulation to better understand how the condition affects the neuro biochemistry.

## Materials and methods

### Patients and healthy controls

Institutional Ethics approval was received from Queensland Metro North and Metro South Human Research Ethics Committees, the Hunter New England Area Health Ethics, and the Australian Defence Health Research Ethics Committee. All patients and controls provided written informed consent prior to commencement of the study.

For this pilot study, we recruited 20 participants, 10 adults with PTSD, and 10 healthy controls, from a number of sources including newspaper advertisement, local psychiatrists, and psychologists. PTSD subjects were eligible if they had been diagnosed with PTSD according to the DSM-V using the Clinician-Administered PTSD scale (CAPS)^27^ and were aged between 18 and 65 years. Exclusion criteria included: current substance use disorder; lifetime history of substance use disorder; current or past history of schizophrenia, bipolar or other psychotic disorder; major head injury, current or past history of neurological disease; current pregnancy or contraindication to MRI scanning. Healthy control participants were included if they were aged between 18 and 65 years and had no current DSM-V Axis I disorder, as assessed by the Structured Clinical Interview for DSM-V (SCID)^28^ and no lifetime history of a mood or anxiety disorder, major head injury, current or past history of neurological disease; current pregnancy or contraindication to MRI scanning. The groups were matched for age, gender and education as shown below in Table 1. Level of education was self-reported as part of the web-based neuro cognitive assessment described below.

**Table 1:** Demographic and clinical characteristics of the PTSD and healthy control participants

Characteristic	PTSD (*n* = 10)	Control (*n* = 10)
Age (years)	44.7 ± 11.36	44.4 ± 11.08
Education (years)	14.4 ± 2.4	15 ± 2.9
Sex (female)	5 (50%)	5 (50%)
CAPS—total	38.4 ± 10.45	
CAPS—intrusion	8.4 ± 0.95	
CAPS—hyperarousal	11.8 ± 1.11	
CAPS—avoidance	5.4 ± 0.48	
*Medications*		
Clonidine	2	
Prazosin	2	
Propranolol	1	
Sertraline	1	
Desvenlafaxine	1	
Escitalopram	1	
Amitriptyline	2	
Venlafaxine	1	

Mean ± SD or *N* (%)

*CAPS* Clinician administered PTSD scale

### Self-report measures and clinical interviews

A clinical psychologist interviewed all participants and administered the CAPS, SCID/IP, Life Events Checklist (LEC)^29^, and Ohio State TBI Identification questionnaire. Index trauma was determined as part of the CAPS assessment. All participants completed a web-based neuro cognitive assessment that quantifies the following domains of cognitive function: sensorimotor, memory, executive planning, attention, and emotion perception (social cognition)^30^. Participants’ current medications were recorded at the time of interview with a clinical psychologist.

### Magnetic resonance imaging and spectroscopy

All scans were performed on a 3 T Prisma scanner (Siemens, Erlangen, Germany, software version VD13D and VE11C) with a 64-channel head and neck coil (Siemens, Erlangen) at one of two sites: Hunter Medical Research Institute (NSW, Australia) or the Herston Imaging Research Facility (QLD, Australia).

### Structural imaging

A three-plane localizer image was performed for volume of interest placement.

After global shimming, a 3D T1-weighted magnetization-prepared rapid gradient-echo (MPRAGE) was acquired (TR/TE/TI = 2530/3.5/1100 ms, flip angle = 7°, field of view = 256 × 256 mm, voxel size 1 × 1 × 1 mm3, IPAT = 3, acquisition time 4:28 min) that was used for MRS voxel placement and whole-brain morphometry. The T1 3D-MPRAGE was reconstructed in sagittal and coronal planes with 1 mm slice resolution for accurate localization of MRS voxel.

### Localized COSY 2D MR spectroscopy

L-COSY data were acquired from a 3 × 3 × 3 cm^3^ voxel positioned in the PCG. L-COSY was acquired with the following parameters: RF carrier frequency at 2.3 ppm; TR 1.5 s; water suppression using WET; 96 t1 increments; with 8 averages per increment, acquired vector size 1024 points; acquisition time 512 ms; spectral width in F2 2000 Hz, and spectral width in F1 1250 Hz (0.8 ms increment size). Time of acquisition was 19 min. To minimize participant motion, soft padding was placed between the participants head and the head coil. Localized shimming was undertaken by adjustment of zero-order and first-order shim gradients using the automatic B0 field mapping technique supplied by the vendor (Siemens AG) followed by manual adjustment of accessible shim gradients to achieve a resulting peak width of water at half-maximum that was 15 Hz or less.

### Evaluation of the COSY data

All participants had satisfactory L-COSY data for analysis. Raw L-COSY data were transferred to MATLAB^31^ for signal combination from multiple elements followed by row concatenation into a 2D matrix. Commercial 2D spectral processing software (Accelrys Felix NMR, 2007) was used for observer-independent spectral processing and measurement of cross and diagonal peak volumes. The processing parameters used were: F2 domain (skewed sine-squared window, 2048 points, magnitude mode) and F1 domain (sine-squared window, linear prediction to 96 points, zero-filling to 512 points, magnitude mode). The measurement of diagonal and cross-peak volumes was performed using a prescribed box, overlying the area of interest. Each box is tailored in size to limit any contamination from surrounding peaks. The volume of signal within the box was measured using Felix NMR^32^. The same area was used for each box to minimize variability. No additional water removal was applied in Felix as water was sufficiently suppressed during acquisition. The total creatine methyl diagonal resonance at 3.02 ppm was used as an internal chemical shift reference in F1 and F2. All crosspeaks are reported as F2-F1 peaks in parts per million.

### Statistical analysis

Mean peak volumes for each test group were compared using both the Student’s *t*-test (two-tailed *P* < 0.05). As the distribution of some metabolites exhibit partial deviation from normality, we also chose to perform Mann–Whitney tests as a non-parametric validation (*P* < 0.05). Correlation analyses between metabolites and clinical variables were performed using Spearman’s correlation (*P* < 0.05). Metabolites were tested for equal variance using the Levene test.

## Results

### PTSD and healthy volunteer cohorts

The PTSD participants reported the following types of trauma: occupational traumatic exposure in emergency services (*n* = 2); occupational traumatic exposure in the police force (*n* *=* 5); deployment related (*n* = 1), life threatened (*n* = 1), and traumatic relationship (*n* = 1). Nine PTSD participants had current major depressive disorder and one had past major depressive disorder (MDD). Two PTSD participants were not on medications, the medications being taken by the remaining participants are detailed in Table 1. None of the healthy volunteers exhibited any PTSD symptoms and had no lifetime history of a mood or anxiety disorder, major head injury, current or past history of neurological disease. Nor were the control cohort found to exhibit any other types of neurological conditions. There was no significant difference between the mean group age, gender, or years of education as shown below in Table 1.

### 2D-COSY

**Fig. 1:**
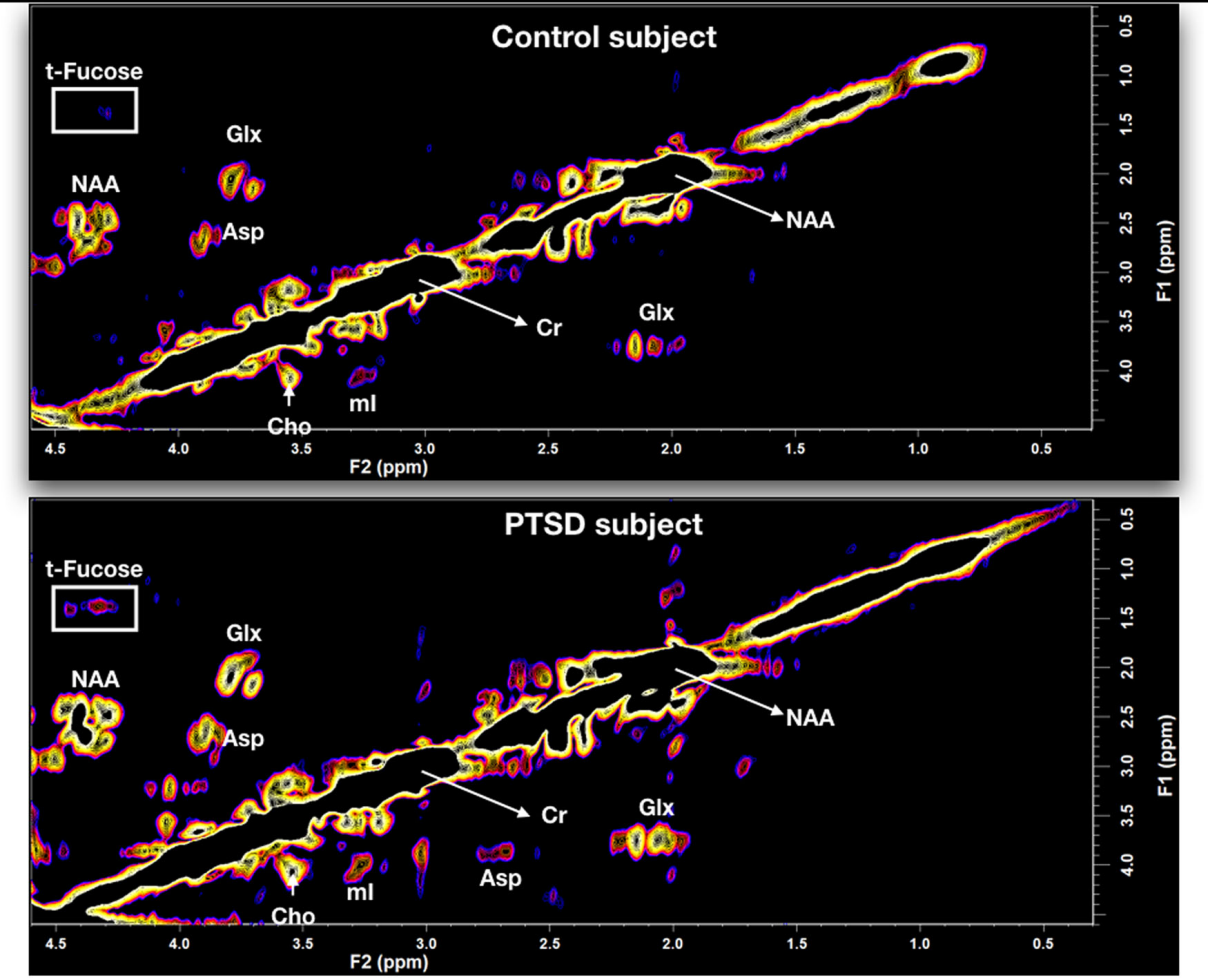
In vivo localized correlated spectroscopy (L-COSY) of the human brain (posterior cingulate gyrus) acquired at 3 T using a 64-channel head coil; voxel size, 30 × 30 × 30 mm^3^; increment size, 0.8 ms; increments, 96; 8 averages per increment; TR =  1.5 s; total experimental time: 19 min; acquired vector, 1024 points; spectral width in F2, 2000 Hz; spectral width in F1, 1250 Hz. Abbreviations: t-Fucose, total-Fucose; Asp, aspartate; Cho, choline; Cr, creatine; Glx, glutamate and glutamine together; m-Ino, myo-inositol; NAA, *N*-acetylaspartate. **The region highlighted by the white box is expanded in Fig.** 2 **. Upper spectrum—healthy control, lower spectrum—participant with PTSD**

**Fig. 2:**
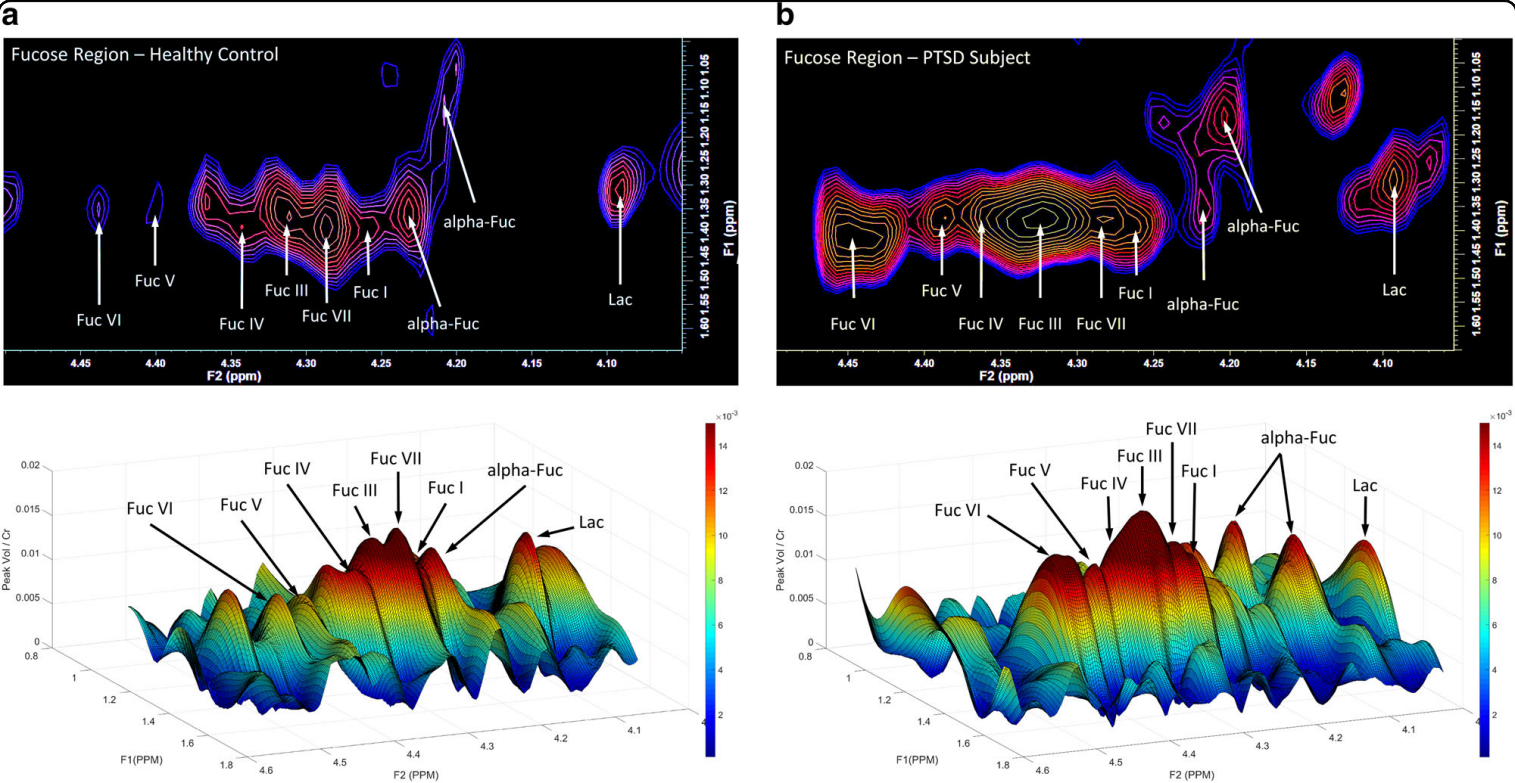
Expanded region (F2, 4.05–4.50 ppm; F1, 1.0–1.65 ppm) of the localized correlated spectroscopy (L-COSY) in Fig. 1 with assignments of fucose I (Fuc I) to fucose VII (Fuc VII) and α-L-fucose denoted^15^. **a** Healthy control where the upper contour plot is produced using Felix software, below this is a three-dimensional plot of the same dataset produced using MATLAB. **b** Data from a typical PTSD patient presented as described above

**Fig. 3:**
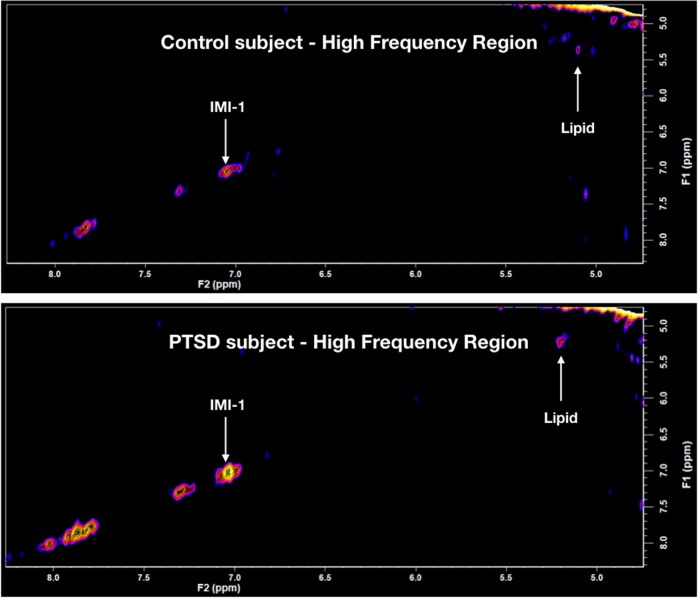
The high- frequency region of in vivo localized correlated spectroscopy (L-COSY) of the human brain (posterior cingulate gyrus) acquired at 3 T using the parameters given in Fig. 1, where both the IMI-1 and double bond of the lipid acyl chain are seen to increase in PTSD. Upper spectrum—healthy control, lower spectrum—participant with PTSD. Abbreviations: IMI-1, imidazole from histamine, histadine, and homocarnosine

A typical 2D-COSY spectrum for a healthy control and a PTSD participant is shown in Figs 1 and 3. The spectral region containing the expanded total fucose region (F2, 4.05–4.50 ppm; F1, 1.0–1.65 ppm) is shown in Fig. 2. A summary of the statistically significant neurochemical differences is shown in Table 2. Statistically significant increases were recorded in IMI-1 (imidazole from histamine, histadine, and homocarnosine) of 12%; total fucose region of 31%; Fuc IV of 48%; Fuc VI of 41%; and an increase in the lipid cross-peak B, which is the HC=CH–CH_2_–CH_2_–CH_3_ of lipid fatty acyl chain of 12.5%. No significant differences were recorded for NAA, glutamine, glutamate, myo-inositol, or GABA using the 2D-L-COSY method.

**Table 2:** Neurochemical changes comparing the PTSD cohort with a healthy age-matched cohort (mean ± SD) in the posterior cingulate cortex identified with 2D L-COSY

Metabolite	Control	PTSD	Average chemical shift (F2-F1) ppm	% Change
IMI-1	2.66 ± 0.22	3.21 ± 0.44**^,a^	7.07– 7.07	+12
t-fucose	5.83 ± 1.36	7.62 ± 1.34**^,a^	3.95–4.50 to 0.90– 1.70	+31
Fucose IV	0.46 ± 0.12	0.69 ± 0.18**^,a^	4.36 – 1.35	+48
Fucose VI	0.43 ± 0.09	0.61 ± 0.19*^,a^	4.45 – 1.35	+41
Lipid HC=CH–CH_2_–CH_2_–CH_3_	5.09 ± 0.55	6.38 ± 1.26**^,a^	1.37–1.99	+13

Significance shown is derived using a 2 T Students *t*-tests with *P < 0.05, **P < 0.01.

*IMI-1* imidazole from histamine, histadine, and homocarnosine

^a^Indicates significance (*P* < 0.05) using Mann–Whitney *U* tests

### Correlation of metabolites with clinical measures

**Fig. 4:**
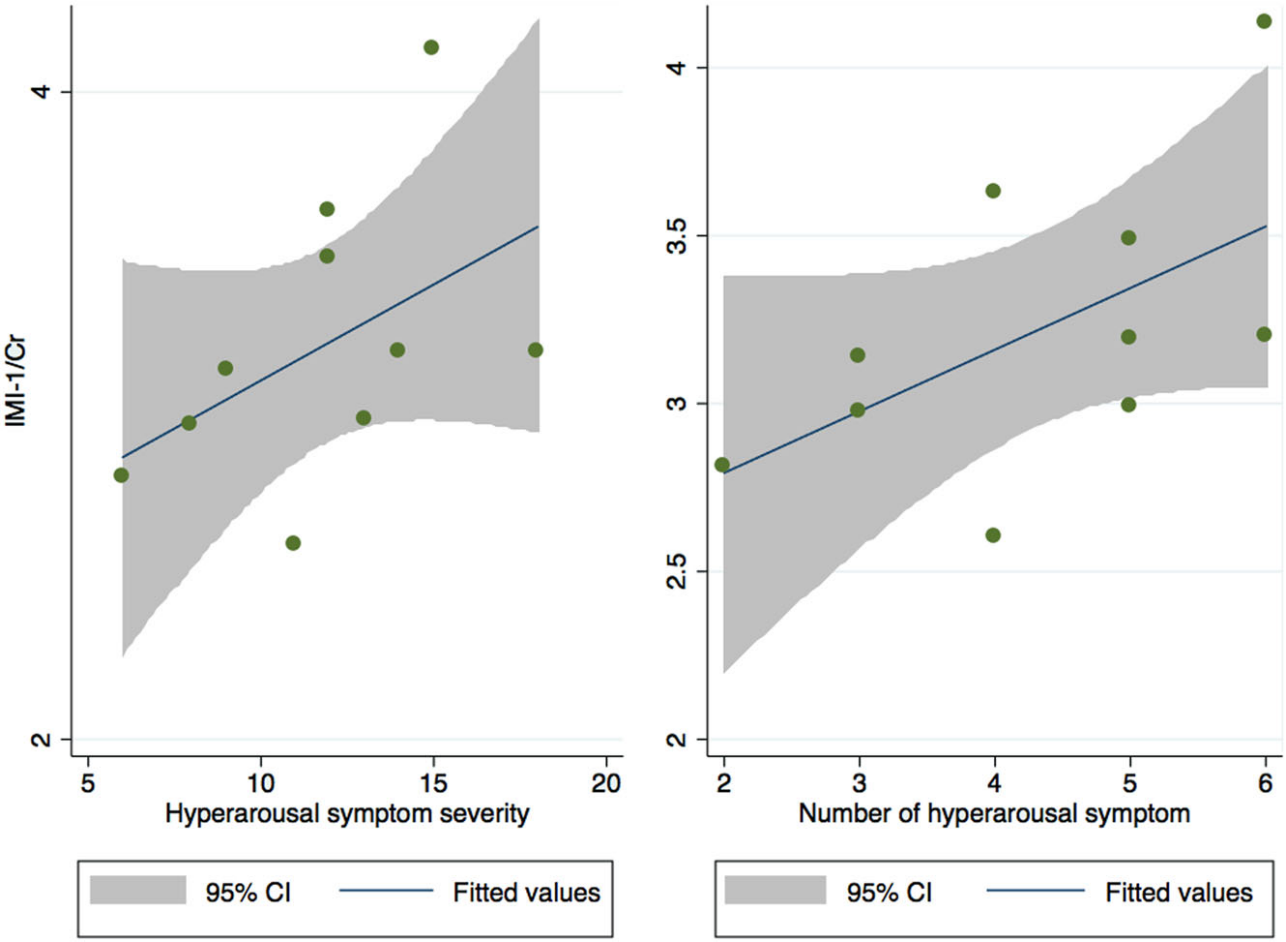
Correlation between the hyperarousal symptoms recorded for the PTSD subjects with the ratio IMI-1/Cr in the PCG region of the brain. Abbreviations: IMI-1: imidazole from histamine, histadine, and homocarnosine

The only statistically significantly different metabolite that correlated with clinical symptoms was IMI-1. Levels of IMI-1 in the PCG were significantly positively correlated with hyperarousal and reactivity (CAPS E symptoms) symptoms and severity (*r* = 0.64, *P* = 0.04 and *r* = 0.66, *P* = 0.04, respectively), shown below in Fig. 4.

## Discussion

The 2D-L-COSY method has identified increases in multiple neurochemicals in the PCG region of the human brain in people suffering from PTSD. The total fucose spectral region, as seen in Fig. 2, was increased by 31%. Two of the fucose-α(1–2)-glycans assigned thus far^15^, Fuc IV, was elevated by 48% and Fuc VI was elevated by 41%. The composite imidazole diagonal resonance of histamine, histidine, and homocarnosine was increased by 12% and the HC=CH–CH_2_–CH_2_–CH_3_ of the MR visible lipid fatty acyl chain is increased by 13%. It is, however, not possible to determine whether the recorded change in the acyl chain is because of alterations in the triglyceride, cholesterol ester or ether-linked fatty acyl chain population, or a mixture thereof. Lipids are normally membrane bound and are not typically visible within the MR spectrum unless there is abnormal pathology^33^.

It is our hypothesis that the COSY method is providing the capacity to monitor the interaction and communication between nerve cells at the level of atoms and molecules. There are two main fields of work supporting this hypothesis.

The first is from the NMR and glycobiology community, who report that according to the positioning of the fucose on the oligosaccharide chain and the saccharide composition of that chain surrounding the fucose, the MR recorded chemical shift will be different, subtly reflect these differences, and be unique^34–36^.

At this period in time, there are five such fucosylated molecules available for inspection in-vivo in the human brain. This means that we are currently recording five fucose molecules each on a different type of oligosaccharide chain. How then can we interpret these results in light of the models proposed by the Hsieh-Wilson lab to explain changes in the fucose-α(1–2)-glycans as a consequence of PTSD?

From rat neuronal cultures and immunocytochemistry^37^, the fucosylated glycans are seen at the synapse of the neurons^37^. The fact that the 2D L-COSY method can record a signal from each, means that the fucose moiety is mobile on the MR timescale. This is consistent with this population of fucose-α(1–2)-glycans being on the end of a neuron. However, there may also be other populations that are not visible on the MR timescale.

The Hsieh-Wilson model^38^ suggests that the proteins on neuron “cell A’s” side of the synapse contain fucose, and that this fucose is binding to proteins on the surface of neuron “cell B”, thereby acting as a chemical bridge across the synapse. In doing so they hypothesize that this in turn will activate the cellular machinery in neuron “cell B” and instruct the cell to synthesize more proteins. They envision a positive feedback loop, in which the proteins synthesized in neuron “cell B” are transported to the cell surface, where they interact with the fucose units from neuron “cell A” to stimulate still more protein synthesis. Collectively their data identify important roles for Fuc-α(1–2)Gal sugars in the regulation of neuronal proteins and morphological changes that may underlie synaptic plasticity.

This model is quite plausible but does not address the need for the fucosyl transferase enzymes involvement in the process nor the need for additional fucose substrate to repopulate the neuron “cell A”. Their model is consistent with the observation that fucose levels increase at the synapse with repeated nerve-cell activity in a healthy brain. By changing the concentration of specific molecules at the synapse, it is believed that certain connections between nerve cells are enhanced and grow stronger over time. This latter hypothesis can now be tested.

What therefore can we deduce from how the fucose-α(1–2)-glycans are affected as a consequence of PTSD? Two specific fucosylated glycans (Fuc IV and VI) are elevated by 48 and 41%; and lipid saturation by 12.5%. Thus, at the very least two of the five, fucosylated glycans available for inspection, are overworking on a cell. The increase of lipid saturation suggests that at least some of those over active species are attached to lipid moieties. Our results indicate that two specific fucolsyated glycans, each attached to a different oligosaccharide chain is activated in those with PTSD. The chemical structure of each has yet to be identified. These data also raise the question as to whether the kinetics of the mechanism of fucose transfer can be recorded and measured in vivo and how this difference recorded for PTSD patients differ for other conditions such as chronic pain. Both are under investigation in our laboratory. It is clear that this COSY technology has opened up a new era in our capacity to monitor and the effect of altered neurochemistry in PTSD, and thus neurobiology, in human development and neurological deficits.

This study has several limitations. PTSD can have multiple co-morbidities and we intentionally recruited participants with minimal co-morbidities. Although it could be argued that this approach may not be representative, it has produced a clear and statistically significant result identifying specific deregulations in the human brain PCG in those with PTSD. In addition, the study groups are small and because of the preliminary nature of the study, we chose to report statistical significance without correcting for multiple tests. However, as we tested 66 analytes, there is an elevated chance of false positives and thus these results should be interpreted with some caution. In an attempt to reduce the chance of a type I error, we have only presented results that were significant using both the Mann–Whitney and *t*-tests.

## Conclusion

A pilot study of PTSD patients, primarily with trauma resulting from occupational traumatic exposure in emergency services and the police force, were evaluated and compared with age-matched and gender-matched healthy controls using in vivo neuro 2D MR spectroscopy in a clinical 3 T MR scanner. The 2D MRS method identified specific neurochemical changes not previously recorded. This includes an increase in two fucose-α(1–2)-glycans as well as the appearance of the substrate α fucose. The fucose-α(1–2)-glycans have been implicated, by others using animal models, in the molecular mechanisms that underlie neuronal development, learning and memory. This is the first evidence of fucose-α(1–2)-glycan involvement in the PTSD pathogenesis in the human brain. Other differences recorded include an increase in imidazole from either histamine, histidine or homocarnosine and an increase in the level of unsaturation in a lipid fatty acyl chain.

## Disclaimer

The work presented in the manuscript has not previously been published or reviewed for publication.
