# Author Correction: Post-traumatic stress disorder affects fucose-α(1–2)-glycans in the human brain: preliminary findings of neuro deregulation using in vivo two-dimensional neuro MR spectroscopy

**DOI:** 10.1038/s41398-019-0400-2

**Published:** 2019-02-05

**Authors:** Scott Quadrelli, Nathan Tosh, Aaron Urquhart, Katie Trickey, Rosanna Tremewan, Graham Galloway, Lisa Rich, Rodney Lea, Peter Malycha, Carolyn Mountford

**Affiliations:** 1 Translational Research Institute, Woolloongabba, QLD 4024 Australia; 2 Center for MR in Health, University of Newcastle, Newcastle, NSW 2308 Australia; 3 Institute of Health and Biomedical Innovation, Queensland University of Technology, Brisbane, QLD 4000 Australia; 4 Radiology Department, Princess Alexandra Hospital, Woolloongabba, QLD 4024 Australia

**Correction to: Translational Psychiatry** **;**

10.1038/s41398-018-0365-6;

Published online 18 January 2019

The original article contained errors in the Fig. 1 caption. The incorrect sentence, “The region highlighted by the white box is expanded in Fig. 3” was corrected to, “The region highlighted by the white box is expanded in Fig. 2.” This has been corrected in the HTML and PDF of the article.

